# Intravenous leiomyomatosis involving the inferior vena cava and right atrium with postoperative pulmonary embolism: a case report

**DOI:** 10.3389/fsurg.2025.1692371

**Published:** 2026-01-06

**Authors:** Yukai Chen, Wangwei Zhang, Aobo Zhuang, Jiaqing Liu, Qinlei Wang, Xiaogang Xia, Wen'gang Li

**Affiliations:** 1 Cancer Research Center, School of Medicine, Xiamen University, Xiamen, China; 2 Department of Hepatobiliary Surgery, Xiang'an Hospital of Xiamen University, Xiamen University, Xiamen, China; 3 Department of Pharmaceutical Toxicology, School of Pharmacy, China Medical University, Shenyang, China; 4 Department of Radiation Oncology, The First Affiliated Hospital of Xiamen University, Xiamen, China

**Keywords:** intravenous leiomyomatosis (IVL), inferior vena cava (IVC), right atrium, pulmonary embolism, multidisciplinary approach

## Abstract

Intravenous leiomyomatosis (IVL) is a rare subtype of benign smooth muscle tumor that can exhibit malignant-like biological behavior. Originating in the uterus, IVL grows in mass-like formations within small veins and can extend into the inferior vena cava (IVC), right atrium, and pulmonary arteries. Due to the potential for life-threatening consequences when IVL invades the heart and pulmonary arteries, early diagnosis and radical resection through a multidisciplinary approach are crucial. This report presents a case of uterine intravenous leiomyomatosis involving the IVC and right atrium, complicated by massive pulmonary embolism after surgery, and includes a literature review to discuss the clinical implications.

## 1 Introduction

Intravenous leiomyomatosis (IVL) is a rare form of smooth muscle tumor characterized by benign histopathology, yet it may exhibit malignant biological behavior. There is no exact data on the prevalence of IVL, while studies have reported that 30% to 80% of fibroids cases are accompanied by extrauterine involvement, and 10% to 30% can spread to the heart (1). When it invades the right ventricle, it can lead to symptoms such as palpitations and dyspnea. In severe cases, it may obstruct the tricuspid valve, the right ventricular outflow tract, or the pulmonary artery, potentially resulting in heart failure or sudden death. Due to the lack of distinctive clinical manifestations and the atypical imaging characteristics associated with IVL, misdiagnosis and missed diagnoses are common. Therefore, the early identification of this condition and accurate assessment are especially critical. This paper reviews a case of IVL that invaded the inferior vena cava and right atrium, culminating in the successful surgical excision of the entire tumor through a multidisciplinary approach, alongside a review of relevant literature.

## 2 Case description

**Figure 1:**
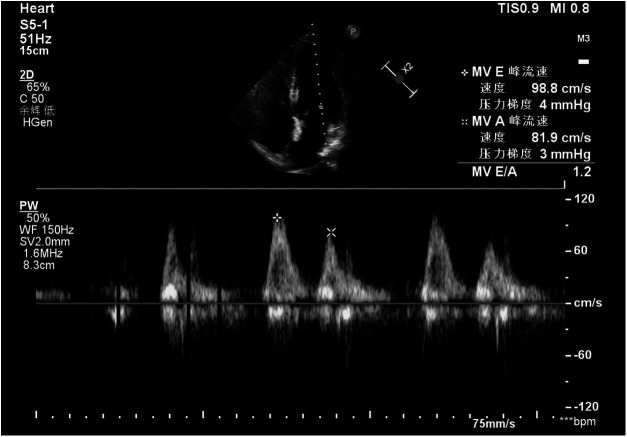
The echocardiographic image of the patient reveals a linear mass within the right atrium.

**Figure 2:**
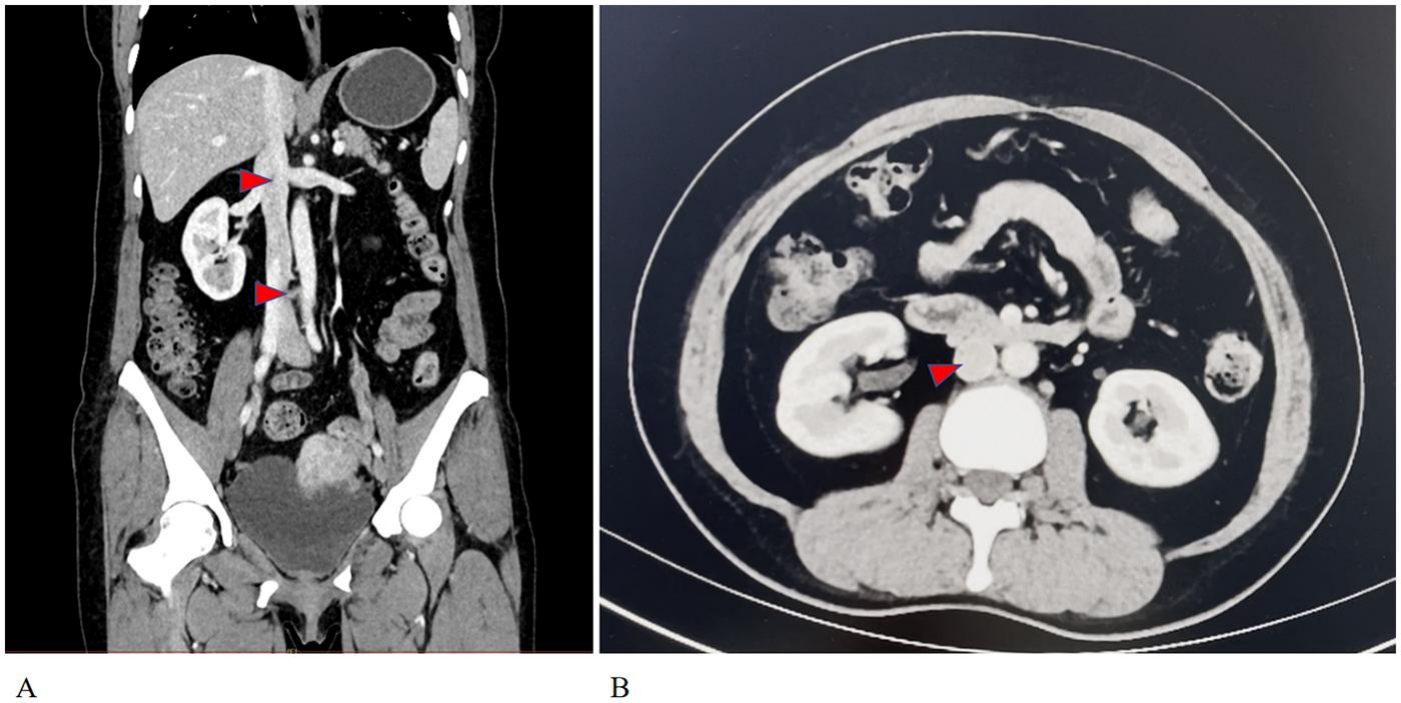
**(A)** the chest enhanced CT imaging in the arterial phase shows a low-density, linear mass indicated by the red arrow, which is not connected to the wall of the vein. **(B)** The red triangle illustrates an intravascular mass within the inferior vena cava that does not enhance in the venous phase.

The patient is a 52-year-old woman who was admitted in October 2024, presenting with the finding of a mass in the major cardiac vessels one month prior. A month before admission, an echocardiogram conducted at a local hospital in Shanxi revealed a space-occupying lesion in the right atrium and inferior vena cava, exhibiting motion in accordance with blood flow. At the time, the patient reported no significant discomfort and sought surgical intervention at Xiang'an Hospital of Xiamen University. The patient had no notable medical history and occasionally consumed alcohol. She experienced menopause at the age of 50. No obvious family history was provided. Gynecological examination indicated that the patient's uterus was consistent with a three-month pregnancy. Doppler ultrasound of the inferior vena cava revealed a band-like structure in the right heart and inferior vena cava, moving with blood flow. Echocardiography showed a band-like echogenic region within the right atrium (Figure 1). The ejection fraction (EF) was measured at 62%, while E/A ratio was 0.9; and pulmonary artery systolic pressure was 53 mmHg, indicating near-normal cardiac diastolic and systolic function. Gynecological B-ultrasound suggested possible adenomyosis, with multiple intramural fibroids in the uterus, and a low echogenicity band-like structure on the right side. Enhanced chest and abdominal CT scans demonstrated a low-density, thread-like lesion extending from the inferior vena cava to the right atrium, not connected to the venous wall, with no enhancement during the venous phase (Figure 2). A MRI scan indicated an occupying lesion within the uterus, possibly representing uterine fibroids. Laboratory tests and electrocardiograms did not reveal any significant abnormalities.

Upon admission, thorough preoperative examinations were conducted, and a multidisciplinary diagnostic and therapeutic approach was initiated. In terms of the surgical plan, the Urologist took the lead in performing bilateral ureteral stenting, while General Surgery focused on addressing the inferior vena cava thrombus. Gynecology would incise the uterus and adnexa, and Cardiothoracic Surgeons ensured readiness for thoracotomy and cardiopulmonary bypass. The Blood Transfusion and Nutrition departments provided perioperative support. On the 13th day of admission, the patient underwent an exploratory laparotomy involving inferior vena cava thrombectomy, bilateral ureteral stent placement, and radical hysterectomy with bilateral adnexectomy. Intraoperatively, no uterine fibroid lesions or ascites were found in the abdominal and pelvic cavities. After appropriately occluding the iliac and lumbar veins, the inferior vena cava wall was opened below the renal vein, and under esophageal ultrasound monitoring, the thrombus in the IVC and right atrium was successfully extracted. A radical resection of the uterus and bilateral adnexa followed to eradicate the uterine fibroid lesions. The surgery lasted 240 min, with a total blood loss of 500 mL and no blood transfusion required.

**Figure 3:**
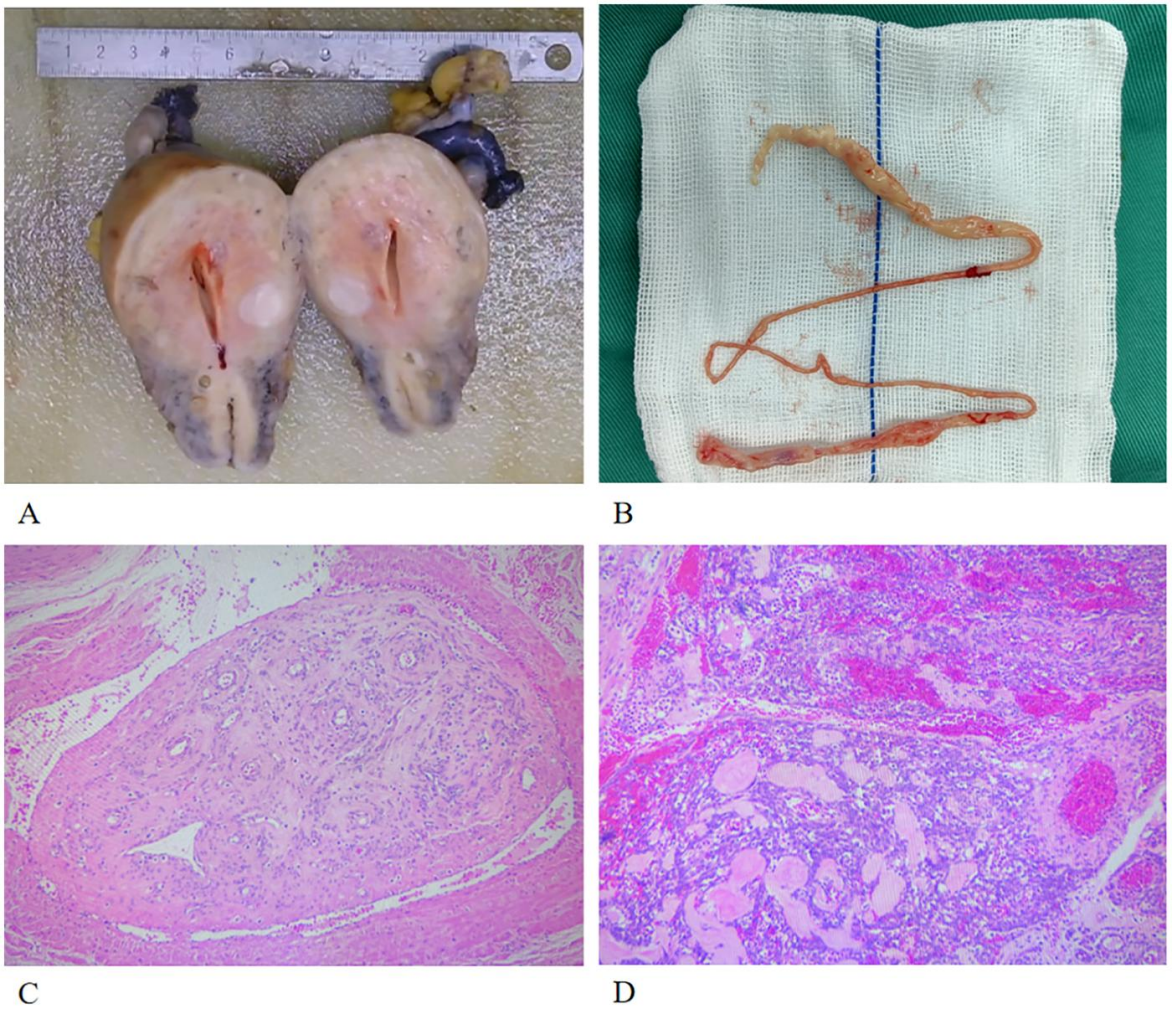
**(A)** The gross specimen of the patient's uterus (with the blue triangle indicating a fibroid); **(B)** The gross specimen of the thrombus in the inferior vena cava; **(C)** The histological specimen of the thrombus in the inferior vena cava; **(D)** The histological specimen of the uterine fibroid.

Postoperative histopathological examination (Figure 3) revealed that the mass from the IVC and right atrium was leiomyomatous tissue (Figure 3B), while multiple intramural and subserosal leiomyomas with angiomyoma were found in the uterus (Figure 3A). Immunohistochemistry (IHC): SMA (+), Caldesmon (focally+), Ki-67 (approximately 2% +).

On the second postoperative day, at around 18:40, the patient experienced dizziness after ambulating, with a drop in oxygen saturation to 60%–70%, while other vital signs remained stable. An urgent pulmonary CTA indicated multiple pulmonary emboli, and a bedside Doppler ultrasound of the lower extremities revealed thrombosis from the right femoral vein to the external iliac vein. The on-call physician rapidly activated the Venous Thromboembolism Multidisciplinary Team (VTE-MDT) protocol, consulting with the Pulmonary Medicine, ICU, and Ultrasound departments. The patient was administered subcutaneous bemiparin for anticoagulation and underwent urokinase thrombolysis. By 19:20, the patient's oxygen saturation improved to 90%–95%.

The patient subsequently made a good recovery and was discharged on the 19th postoperative day without the use of anti-estrogen medications. A follow-up examination six months later indicated that the patient was in good general health, with no signs of tumor recurrence detected.

## 3 Discussion

The pathogenesis of IVL remains uncertain, with two prevailing theories acknowledged by most scholars: 1) IVL originates from uterine smooth muscle tumors and infiltrates the venous lumen; 2) IVL originates from smooth muscle cells within the venous wall. A retrospective study by Peng et al. (2) indicated that 87.3% of IVL patients had clear evidence of intramural origin within the uterine muscle wall, and 46.5% had a history of uterine myomectomy or cesarean section. Immunohistochemical research has demonstrated that IVL cells often exhibit positive staining for Desmin, SMA, estrogen, and progesterone receptors (3), while these factors are often negative or weakly positive in the smooth muscle cells of the vascular wall. These findings support the first theory.

IVL can be classified into four stages based on the extent of involvement and tumor morphology: Stage 1 involves thrombosis limited to the veins within the uterine muscle layer, extending to the iliac vein or ovarian vein/renal vein, but not entering the inferior vena cava; Stage 2 involves the tumor extending to the inferior vena cava but not affecting the heart; Stage 3 involves further extension of the tumor to the right atrium but not reaching the pulmonary artery; Stage 4 involves invasion of the pulmonary artery and/or metastasis to the lungs. For Stage 2 and above, IVL is further classified into three types based on tumor morphology: Type I entails continuous thrombus, with the intravenous lesions not adhering to the vascular wall; Type II involves thrombus adhering to the vascular wall or cardiac structures; and Type III consists of pedunculated lesions attached to the vascular intima or endocardium (4). Based on preoperative imaging and ultrasound examinations, the IVL thrombus in this case extended upwards to the right atrium, exhibiting mobility with the bloodstream, and there was no evidence of adherence to the vascular wall or cardiac tissue. Therefore, this case can be categorized as Stage 3, Type I.

Largely depending on the extent of the lesions, Clinical manifestations of IVL is hardly specific. In the early stages, it often presents common symptoms of uterine fibroids, such as abnormal uterine bleeding, pelvic masses, abdominal pain, and bloating (5). When the lesions involve the IVC, lower limb edema and Budd-Chiari syndrome may occur. Invasion into the heart and pulmonary artery can lead to chest tightness, dyspnea, syncope, heart failure, or even sudden death (6). Moreover, 12%–36% of IVL patients are asymptomatic (4), seeking medications simply due to uterine fibroids or cardiac vascular occupancy. In the present case, the patient visited the hospital due to the discovery of occupying lesions in the right atrium and IVC during a routine physical examination without any discomfort. On admission, gynecological consultation and ultrasound examination ultimately confirmed the diagnosis of uterine fibroids, delineated the nature of the cardiovascular occupancy, and ultimately, it was pathologically confirmed after surgery. Therefore, enhancing clinical awareness of IVL is crucial. When patients with lower vena cava or cardiac lesions accompanied by a history of uterine occupancy or uterine fibroids, or when uterine fibroid patients present with chest tightness, dyspnea, or lower limb swelling, clinicians in relevant departments should be vigilant to the possibility of IVL and promptly involve the gynecologists to establish a diagnosis and treatment plan.

Surgical removal remains the preferred treatment for IVL patients without contraindications. Bilateral oophorectomy is considered to reduce tumor recurrence. For patients with tumor extension to the inferior vena cava and heart, the recommended surgical approach is radical excision of the uterus, bilateral ovaries, and all tumors (7). However, some studies suggest that the recurrence and progression of IVL are not related to oophorectomy and advocate for preserving the ovaries whenever possible (4, 8). As the tumor spreads along the venous system to the right heart and even the pulmonary artery, over 60% of Stage 3 and almost all Stage 4 IVL cases historically required surgical intervention via open-chest or thoracoabdominal approaches under cardiopulmonary bypass (4). Nevertheless, with advancements in surgical techniques, the likelihood of avoiding open-chest procedures and cardiopulmonary bypass has been increasing for Type I IVL patients ranked Stage 2 and above. In this case, the patient falls under Stage 3 Type I IVL. Preoperative examinations did not confirm any evidence of the thrombus adhering to the vascular wall or cardiac tissue, leading to a smooth surgical procedure without the utilization of cardiopulmonary bypass.

IVL patients should take precautions to prevent perioperative venous thromboembolism (VTE). The main causes include hemodynamic disturbances due to major vessel incisions during surgery, prolonged bed rest before and after surgery, and the surgery itself increases the risk of postoperative bleeding. Therefore, perioperative VTE prevention for such patients should primarily focus on mechanical prevention, such as the use of elastic stockings before and after surgery and the placement of an IVC filter during surgery. Additionally, in rare cases, IVL thrombi entering the pulmonary circulation can also act as emboli causing pulmonary embolism. We retrieved two case reports (9, 10) of IVL-related NTPE from the past 10 years and found that both cases were admitted to the hospital due to exertional dyspnea lasting for several weeks. They had no previous risk factors for DVT and were ultimately diagnosed due to ineffective anticoagulant or thrombolytic therapy. It should be noted that since acute PE is life-threatening and typically responds to thrombolysis or anticoagulation, a diagnosis of IVL-related NTPE is often one of exclusion, made only after the failure of emergency anticoagulant or thrombolytic therapy. In our case, the patient responded well to anticoagulant and thrombolytic therapy. Taking into account other examination results, we tend to believe that the cause of the patient's PE was the migration of a lower limb venous thrombus.

Currently, there is still debate regarding whether IVL requires anti-estrogen therapy. Presently, anti-estrogen therapy is mostly used as an adjuvant treatment during the perioperative period to prevent the progression and recurrence of IVL, and to increase the progression-free survival period for patients with incomplete removal of IVL tumors. Optional medications include gonadotropin-releasing hormone agonists (GnRH-a), aromatase inhibitors, and tamoxifen (11). However, studies have also indicated that there is no significant benefit from anti-estrogen treatment for patients with complete removal of IVL tumors, and aromatase inhibitors may even promote recurrence in patients who have completely removed the tumors (4). In this case, the patient underwent bilateral oophorectomy and radical excision of all tumor lesions during surgery, thus obviating the need for anti-estrogen medication.

Research indicates a recurrence rate of 9.7% for Stage 1 IVL patients with complete lesion excision, while 39% of those who were unable to completely remove the lesion experienced residual lesion progression after surgery. The factors influencing IVL recurrence and progression exhibit significant variations among different studies, involving age, surgical approach, tumor size, and drug therapy (3, 4, 11, 12). Therefore, long-term follow-up is recommended for IVL patients, with a follow-up interval of every 3–6 months within 2 years after surgery, followed by semi-annual to annual follow-ups thereafter (7). Imaging examinations are the primary method for follow-up.

## 4 Conclusion

Intravascular leiomyomatosis is a rare benign tumor predominantly observed in women of reproductive age and those in the perimenopausal stage. Early detection and prompt intervention are crucial for accurate diagnosis, successful surgical outcomes, and the prevention of recurrence. Throughout the patient care procedures, multidisciplinary collaboration is essential. Comprehensive assessments, considering factors such as the patient's age, fertility desires, and the extent of the lesions, should guide the formulation of personalized surgical plans. This approach aims to achieve radical tumor resection while maximizing the patient's quality of life.

## Data Availability

Due to privacy and ethical restrictions, the raw data supporting this case report (e.g., patient medical records, original imaging files) are not publicly available. De-identified data may be obtained from the corresponding author upon reasonable request, subject to compliance with ethical approval and the scope of the patient's informed consent.

## References

[1] Liu B Liu C Guan H Li Y Song X Shen K Intravenous leiomyomatosis with inferior vena cava and heart extension. J Vasc Surg. (2009) 50(4):897–902. 10.1016/j.jvs.2009.04.037 19560308

[2] Peng J Zhong F Zhu Y Zhang M Zhang M Lu C Clinical analysis of uterine intravenous leiomyomatosis: a retrospective study of 260 cases. J Obstet Gynaecol Res. (2021) 47(12):4357–64. 10.1111/jog.15013 34525488 PMC9293182

[3] Yu X Fu J Cao T Huang L Qie M Ouyang Y. Clinicopathologic features and clinical outcomes of intravenous leiomyomatosis of the uterus: a case series. Medicine (Baltimore). (2021) 100(1):e24228. 10.1097/MD.0000000000024228 33429819 PMC7793403

[4] Wen Y Ma G Miao Q Shao J Lu W Liu X The largest single-center report on intravenous leiomyomatosis and development of a classification to guide surgical management. J Vasc Surg Venous Lymphat Disord. (2025) 13(1):101989. 10.1016/j.jvsv.2024.101989 39395469 PMC11764821

[5] Baxter BL Hur HC Guido RS. Emerging treatment options for fibroids. Obstet Gynecol Clin North Am. (2022) 49(2):299–314. 10.1016/j.ogc.2022.03.001 35636810

[6] Li B Chen X Chu YD Li RY Li WD Ni YM. Intracardiac leiomyomatosis: a comprehensive analysis of 194 cases. Interact Cardiovasc Thorac Surg. (2013) 17(1):132–8. 10.1093/icvts/ivt117 23563052 PMC3686387

[7] Li H Xu J Lin Q Zhao Y Tong H Tu R Surgical treatment strategies for extrapelvic intravenous leiomyomatosis. Orphanet J Rare Dis. (2020) 15(1):153. 10.1186/s13023-020-01394-9 32546179 PMC7296750

[8] Yu X Zhang G Lang J Liu B Zhao D. Factors associated with recurrence after surgical resection in women with intravenous leiomyomatosis. Obstet Gynecol. (2016) 128(5):1018–24. 10.1097/AOG.0000000000001718 27741175

[9] Kloska M Patel P Soliman A Musco K Rovella J. Intravenous leiomyomatosis: an uncommon cause of pulmonary embolism. Am J Case Rep. (2021) 22:e931386. 10.12659/AJCR.931386 34381010 PMC8369432

[10] Wu YH Lee YT Lee CI Tzeng YH Wei J. Nonthrombotic pulmonary embolism caused by intravenous leiomyomatosis: a case report. Medicine (Baltimore). (2019) 98(3):e14118. 10.1097/MD.0000000000014118 30653137 PMC6370129

[11] Ma G Miao Q Liu X Zhang C Liu J Zheng Y Different surgical strategies of patients with intravenous leiomyomatosis. Medicine (Baltimore). (2016) 95(37):e4902. 10.1097/MD.0000000000004902 27631266 PMC5402609

[12] Low HY Zhao Y Huang KS Shen HP Wu PJ Tseng CJ. Intravenous leiomyomatosis of the uterus: a clinicopathological analysis of nine cases and literature review. Taiwan J Obstet Gynecol. (2017) 56:362–5. 10.1016/j.tjog.2017.04.017 28600049

